# Rituximab Infusion Timing, Cumulative Dose, and Hospitalization for COVID-19 in Persons With Multiple Sclerosis in Sweden

**DOI:** 10.1001/jamanetworkopen.2021.36697

**Published:** 2021-12-01

**Authors:** Kyla A. McKay, Fredrik Piehl, Simon Englund, Anna He, Annette Langer-Gould, Jan Hillert, Thomas Frisell

**Affiliations:** 1 Neuro Division, Department of Clinical Neuroscience, Karolinska Institutet, Stockholm, Sweden; 2 Centre for Molecular Medicine, Karolinska University Hospital, Stockholm, Sweden; 3 The Karolinska University Hospital and Academic Specialist Centre, Stockholm Health Services, Stockholm, Sweden; 4 Clinical Epidemiology Division, Department of Medicine, Solna, Karolinska Institutet, Stockholm, Sweden; 5 Department of Neurology, Los Angeles Medical Center, Southern California Permanente Medical Group, Los Angeles

## Abstract

This case-control study examines the risk of severe COVID-19 infection in individuals with multiple sclerosis who were receiving rituximab.

## Introduction

Rituximab therapy has been associated with more severe COVID-19 infection in persons with multiple sclerosis,^1,2^ although this finding was based on a small number of events. Further research is needed to evaluate the potential increased risk for severe disease. In this study, we examined the association between timing and dose of rituximab and hospitalization for COVID-19 across Sweden.

## Methods

This nationwide nested case-control study used prospectively collected data from the COMBAT-MS (Comparison Between All Immunotherapies for Multiple Sclerosis) observational drug trial cohort (eMethods in the Supplement). Participants were persons who were receiving ongoing treatment with rituximab and had COVID-19 onset (reported by a neurologist) between March 1, 2020, and April 30, 2021. This study received approval from the Regional Ethics Review Board in Stockholm. Written informed consent was obtained from all participants. We followed the Strengthening the Reporting of Observational Studies in Epidemiology (STROBE) reporting guideline.

The odds of hospitalization for COVID-19 were related to (1) the time between the most recent rituximab infusion and the COVID-19 onset date (months) and (2) the total cumulative lifetime dose of rituximab received (grams) using logistic regression. The reference cohort included all participants with mild COVID-19 infection, defined as those who did not require hospitalization. Models were adjusted for age, sex, and prepandemic Expanded Disability Status Scale score (range: 0-10, with the highest score indicating greatest level of disability). Data on race and ethnicity were not collected. A sensitivity analysis included only persons with polymerase chain reaction–confirmed COVID-19 infection.

Findings were reported as odds ratios (ORs) with 95% CIs. A 2-sided *P* < .05 indicated statistical significance. Pearson χ^2^ or Fisher exact test was used for categorical variables, whereas unpaired, 2-tailed *t* test or Mann-Whitney test was used for continuous variables. SAS, version 9.4 (SAS Institute), was used for all statistical analyses.

## Results

Of the 3391 persons who were enrolled in the COMBAT-MS cohort, 326 (9.6%) contracted COVID-19 infection during the study period. Among these individuals, 172 (52.8%) received rituximab before COVID-19 onset. Twenty-six persons (15.1%) required hospitalization, 5 of whom were admitted to the intensive care unit and 4 of whom required ventilation. No deaths occurred.

No differences were observed in terms of age, disease duration, or disease course between persons with COVID-19 that required hospitalization and those with a mild case (Table 1). The median (IQR) time between the most recent rituximab infusion and COVID-19 onset was 6.1 (3.9-11.0) months among those with a mild case and was 4.6 (3.6-5.6) months among those who were hospitalized (the difference was not significant at *P* = .16). Persons with mild COVID-19 had a median (IQR) cumulative lifetime rituximab dose of 3.5 (2.5-4.5) grams compared with 3.3 (2.6-4.5) grams for hospitalized cases. Time from the most recent disease-modifying therapy infusion was not associated with the odds of requiring hospitalization (adjusted OR, 0.99; 95% CI, 0.92-1.04) nor was a cumulative lifetime dose of rituximab (adjusted OR, 1.08; 95% CI, 0.83-1.38). After the categorization of rituximab timing, persons with an infusion less than 4 months (vs >8 months) before COVID-19 onset were more likely to be hospitalized, but this association was not significant after adjustment for age, sex, and Expanded Disability Status Scale score. Sensitivity analyses of polymerase chain reaction–confirmed COVID-19 cases revealed comparable results (Table 2).

**Table 1.** Clinical and Demographic Characteristics of the Cohort

Characteristic	COVID-19 cases		*P* value
	Mild	Hospitalized	
No. of cases	146	26	
Demographic and clinical characteristics			
Age, mean (SD), y	41.1 (9.9)	43.0 (10.9)	.37^a^
Disease duration, median (IQR), y	9.0 (5.8-13.7)	7.7 (3.9-15.4)	.35^b^
Sex, No. (%)			
Female	111 (76.0)	13 (50.0)	.01^c^
Male	35 (24.0)	13 (50.0)	
Resident of Stockholm, No. (%)	88 (60.3)	18 (69.2)	.52^c^
Progressive course, No. (%)	5 (3.4)	1 (3.8)	>.99^d^
EDSS score, median (IQR)			
Prepandemic^e^	1.5 (1.0-2.5)	2.0 (1.5-3.0)	.03^b^
Post–COVID-19	1.5 (1.0-2.0)	2.0 (1.9-2.5)	.06^b^
Change in EDSS score	0 (0-0.3)	0 (0-0.5)	.75^b^
Rituximab timing and dose, median (IQR)			
Time between rituximab infusion and COVID-19 onset, mo	6.1 (3.9-11.0)	4.6 (3.6-5.6)	.16^b^
Cumulative lifetime dose of rituximab, g	3.5 (2.5-4.5)	3.3 (2.6-4.5)	.70^b^

Abbreviation: EDSS, Expanded Disability Status Scale.

^a^ Calculated using unpaired, 2-tailed *t* test.

^b^ Calculated using Wilcoxon rank-sum test.

^c^ Calculated using χ^2^ test.

^d^ Calculated using Fisher exact test.

^e^ Most recent score in the prepandemic year (March 1 to February 29, 2020).

**Table 2.** Logistic Regression Models Comparing Hospitalized Cases With Mild COVID-19 Cases by Rituximab Timing and Dose

Variable	COVID-19 cases, OR (95% CI)			
	All (n = 172)		Confirmed (n = 127)	
	Unadjusted	Adjusted^a^	Unadjusted	Adjusted^a^
**Rituximab timing**				
Continuous variable				
Time since most recent rituximab infusion, mo	0.97 (0.90-1.03)	0.99 (0.92-1.04)	0.97 (0.90-1.02)	0.99 (0.92-1.04)
Categorical variable				
Time since most recent rituximab infusion, mo				
<4	3.27 (1.11-10.60)	1.98 (0.60-6.95)	2.78 (0.95-8.86)	1.83 (0.50-7.05)
4-8	2.23 (0.72-7.33)	1.47 (0.42-5.24)	2.36 (0.77-7.72)	1.72 (0.46-6.58)
>8	1 [Reference]	1 [Reference]	1 [Reference]	1 [Reference]
**Rituximab dose**				
Continuous variable				
Cumulative lifetime dose, g	1.05 (0.84-1.31)	1.08 (0.83-1.38)	1.03 (0.82-1.29)	1.02 (0.80-1.32)
Categorical variable				
Cumulative lifetime dose, g				
<3.0	1 [Reference]	1 [Reference]	1 [Reference]	1 [Reference]
3.0-4.5	1.64 (0.59-4.78)	1.53 (0.45-5.43)	1.28 (0.47-3.74)	1.10 (0.32-4.00)
>4.5	1.19 (0.40-3.64)	1.24 (0.36-4.38)	1.06 (0.31-3.60)	0.81 (0.18-3.47)

Abbreviation: OR, odds ratio.

^a^ Analyses were adjusted for age, sex, and prepandemic Expanded Disability Status Scale score.

## Discussion

Among 172 Swedish persons with multiple sclerosis who received rituximab before contracting COVID-19, we found no statistically significant association between the timing of rituximab infusion or cumulative lifetime rituximab dose and the odds of hospitalization for COVID-19, although the study power was limited. Much research has been conducted on the potential risk of severe COVID-19 infection associated with rituximab exposure, but the conclusions have been drawn from small patient populations, and the evidence has been inconsistent overall.^1,2,3,4^ The findings of the present study do not support a strong temporal association between the most recent dose of rituximab and hospitalization for COVID-19; these findings are similar to the results of a study in Italy^1^ but counter those of a study from California.^2^ Both of these previous studies included few cases (<10) with severe COVID-19 infection. Although the present study was larger, its power was limited and thus we were unable to rule out an association of modest-to-moderate size.

The strengths of this study were the prospective collection of data, nationwide study population, and high-quality data.^5^ The limitations included potential ascertainment bias given the awareness of an increased risk of infection associated with rituximab^6^ and missing information on known risk factors for COVID-19 severity, including smoking and comorbidity. In conclusion, large data sets with data capture that is less prone to surveillance bias and with access to a broader range of confounding factors are necessary to settle the question of whether continued use of rituximab during the COVID-19 pandemic increased the risk of severe COVID-19 infection.

## Notes

Supplement. **eMethods.** COMBAT-MS Observational Drug Trial
