# Supplementary material for: Rituximab Infusion Timing, Cumulative Dose, and Hospitalization for COVID-19 in Persons With Multiple Sclerosis in Sweden
Source: JAMA Netw Open. 2021 Dec 1;4(12):e2136697. doi: 10.1001/jamanetworkopen.2021.36697 (PMC8637249; doi:10.1001/jamanetworkopen.2021.36697)
Supplement: Supplement. — eMethods. COMBAT-MS Observational Drug Trial [file jamanetwopen-e2136697-s001.pdf]

Supplementary Online Content

McKay KA, Piehl F, Englund S, et al. Rituximab infusion timing, cumulative dose, and hospitalization for COVID-19 in persons with multiple sclerosis in Sweden. *JAMA Netw Open*. 2021;4(12):e2136697. doi:10.1001/jamanetworkopen.2021.36697

eMethods. COMBAT-MS Observational Drug Trial

This supplementary material has been provided by the authors to give readers additional information about their work.

© 2021 McKay KA et al. *JAMA Network Open.*

eMethods. COMBAT-MS Observational Drug Trial

The COMBAT-MS observational drug trial is a nationwide Swedish observational drug trial aiming to compare the safety and effectiveness of all disease modifying therapies (DMTs) used to treat MS in clinical praxis, and aimed to enroll all MS patients starting a new DMT between 2011 to 2018 at a Swedish University clinic, with prospective data capture through 2021. The data contain detailed demographic and disease-specific information, including MS onset date, disease course, DMT exposure (date and dose of all infusions), and Expanded Disability Status Scale (EDSS) scores. COVID-19 information (date of symptom onset, polymerase chain reaction [PCR] testing, hospitalization, intensive care unit [ICU] admission, ventilation, and death) has been collected since March 2020. Aside from information on death, which was based on linkage with the Swedish Cause of Death Register, all Covid-19 related outcomes were reported by the patient to their neurologist.

Characteristics, were compared between hospitalized and 'mild' cases of COVID-19, not requiring hospitalization, using the Pearson's χ^2 or Fisher's exact test for categorical variables, and the student's t-test or Mann-Whitney U-test for continuous variables. The level of statistical significance was determined *a priori* as < 0.05. This report follows the STROBE reporting guidelines.

© 2021 McKay KA et al. *JAMA Network Open.*
